# BVLab-analyzer: a unified visual toolbox for terabyte-scale whole-brain cell detection, neuron reconstruction, and vascular network extraction

**DOI:** 10.3389/fninf.2026.1925112

**Published:** 2026-10-07

**Authors:** Ying Hu, Xiaojun Wang, Pei Sun, Yuan Shen, Tingwei Quan

**Affiliations:** 1 School of Mathematics and Statistics, Hubei University of Education, Wuhan, Hubei, China; 2 Institute of Big Data Analysis and Applied Mathematics, Hubei University of Education, Wuhan, Hubei, China; 3 Key Laboratory of Biomedical Engineering of Hainan Province, School of Biomedical Engineering, Hainan University, Sanya, China; 4 Department of Clinical Research Institute, Central People’s Hospital of Zhanjiang, Zhanjiang, Guangdong, China; 5 Britton Chance Center for Biomedical Photonics, Wuhan National Laboratory for Optoelectronics-Huazhong University of Science and Technology, Wuhan, Hubei, China

**Keywords:** 3D skeleton reconstruction, brain-wide reconstruction, deep learning, large-scale volume data analysis, zero-code software

## Abstract

BVLab-Analyzer is a standalone graphical software tool for end-to-end analysis of terabyte-scale whole-brain microscopy images. Operating through a zero-code interface, it handles data import (TIFF, BioImageVision, OME-Zarr), deep learning-based semantic segmentation, 3D skeleton reconstruction, and morphological statistical analysis within a unified pipeline. The software supports three target structures—cells, neurons and vasculature—and exports their morphological reconstructions in standard SWC format. To accelerate training data preparation, Smart Annotation tools provide sub-volume interference isolation, coarse-vessel ray tracing, and skeleton auto-interpolation. For inference, optimized block-wise strategies enable a standard 3D U-Net to predict whole-brain volumes up to 12 TB on a single consumer-grade GPU, while an interrupt-and-resume engine ensures fault tolerance during extended computations. By integrating these features, BVLab-Analyzer lowers the hardware barrier for whole-brain analysis and unifies previously fragmented toolchains—from annotation and segmentation to reconstruction and quantification—into a single, accessible software environment.

## 1 Introduction

Advanced light-sheet microscopy, serial two-photon tomography, and fluorescence micro-optical sectioning tomography (fMOST) enable three-dimensional imaging of a single mammalian whole brain, routinely generating raw volumetric data ranging from several terabytes to over 10 terabytes (Hillman et al., 2019; Li et al., 2010; Ragan et al., 2012). Combined with tissue clearing techniques and c-Fos immunofluorescence labeling, these imaging pipelines can map whole-brain neuronal activity atlases at single-cell resolution (Ueda et al., 2020). Imaging throughput is no longer the bottleneck; downstream data processing and volumetric quantification are the core challenges. Converting raw voxels into meaningful morphological parameters, such as cell counts, axonal spatial length, and vascular branch density, requires high-fidelity semantic segmentation and skeleton tracing as the first step.

To address the challenges of whole-brain image analysis, the community has developed a rich ecosystem of tools. ClearMap pioneered automated c-Fos cell detection using classical image processing methods (Renier et al., 2016; Kirst et al., 2020). Cellfinder introduced convolutional neural networks for three-dimensional cell localization (Tyson et al., 2021). DELiVR and NEATmap further leveraged deep learning for whole-brain cell activity mapping (Kaltenecker et al., 2024; Zheng et al., 2024). TrailMap and HP-VSP specialized in curvilinear structures, optimizing the automated segmentation of complex axonal projections and capillary networks, respectively (Friedmann et al., 2020; Li et al., 2023). Among general-purpose tools, nnU-Net provided an automated benchmark for biomedical segmentation (Isensee et al., 2021); Cellpose and StarDist excelled at general cell instance segmentation (Stringer et al., 2021; Schmidt et al., 2018); ilastik offered flexible interactive machine learning (Berg et al., 2019). Vaa3D and BigNeuron established a systematic ecosystem for interactive three- dimensional visualization and neuron reconstruction (Peng et al., 2010; Peng et al., 2015). These works collectively validated the feasibility of deep learning for whole- brain analysis and established methodological benchmarks for subsequent tool development.

Despite significant progress, two practical barriers still hinder the widespread deployment of these tools in routine biology laboratories. First, existing deep learning frameworks typically require multi-GPU clusters or large-memory enterprise-grade servers when processing terabyte-scale data. As noted by Cellfinder, training deep networks on whole-brain volumetric data demands substantial computational resources (Tyson et al., 2021), which effectively places whole-brain analysis beyond the reach of laboratories equipped with only a single consumer-grade GPU. Second, in systems neuroscience, cell somata, neuronal processes, and the microvascular system are not isolated entities but together form the neurovascular unit (NVU). However, existing software tools are specialized for a single morphological type or a single processing step. A laboratory exploring the relationships among these three components of the neurovascular unit must learn multiple software packages, contend with incompatible data formats, and manually bridge disconnected processing pipelines.

To democratize whole-brain data analysis, we developed BVLab-Analyzer, a unified, zero-code Windows GUI that integrates cell detection, neuronal processes reconstruction, and vascular network extraction within a single software environment. The entire tool completes all workflows on a single consumer-grade GPU, bridging the gap between raw voxel segmentation and standardized skeleton reconstruction. To clarify the positioning of BVLab-Analyzer relative to existing tools, Table 1 provides a multi-dimensional capability comparison.

**Table 1:** Tool capability comparison.

Capability	BVLab-analyzer	ClearMap 2.x	Cellfinder	nnU-Net	Cellpose
Target structures	Cell, neuron, vessel in unified pipeline	Cell (CellMap) + vessel (TubeMap), separate modules	Cell only	General-purpose, user-assembled	Cell only
Interface	Zero-code standalone GUI	Python libraries, scripts, CLI	napari/ImageJ plugin	Python, manual configuration	Dedicated GUI (cell-focused)
End-to-end coverage	Import→annotation→training→inference→stitching→SWC → statistics	Modular, no unified zero-code workflow	Single analysis step	Single segmentation step	Single segmentation step
Data scale	0.47–12.36 TB whole-brain	TB-scale (light-sheet/clearing)	Sub-volume, memory-limited	User-managed tiling	Conventional single volumes
Data format	TIFF/BV/OME-Zarr	iDISCO+clearing+light-sheet pipeline	Standard brain imaging	Manual format conversion	Standard imaging
TB-scale tiling/stitching/resume	Built-in automated interrupt-resume	TB-scale stitching, no GUI integration	N/A	N/A, manual stitching	N/A
Hardware	Single consumer GPU	CPU-based, large memory	Single GPU, small volumes	Multi-GPU cluster recommended	Single GPU, small volumes
Post-processing/statistics/SWC	Built-in skeletonization, statistics, SWC export	Vessel graph, no integrated statistics	Limited post-processing	N/A	N/A

## 2 Materials and methods

### 2.1 Architecture and data processing

BVLab-Analyzer is built on a hybrid core of C++ (for compute-intensive operations) and Python (for deep learning logic), wrapped in a Qt graphical interface. The deep learning pipeline uses PyTorch with NVIDIA CUDA acceleration. To avoid environment configuration issues, the software is compiled as a standalone Windows executable, pre-bundled with all runtime binaries and dependencies, eliminating the need for manual Python environment setup.

The application is organized into six core modules within an interactive tabbed panel (Figure 1): Data I/O (format parsing, image pyramid construction), Annotation (interactive sparse labeling), Training (3D U-Net with loss monitoring and checkpointing), Inference (block-wise prediction with interrupt-and-resume), Post-processing (structure-specific skeletonization and SWC export), and Statistics (batch morphological quantification and CSV export). All modules communicate through a central project file (.bvproj), ensuring automatic parameter transfer and fault-tolerant state recovery. The software is distributed as a standalone executable with no CLI or external model support, prioritizing zero-code accessibility.

**Figure 1:**
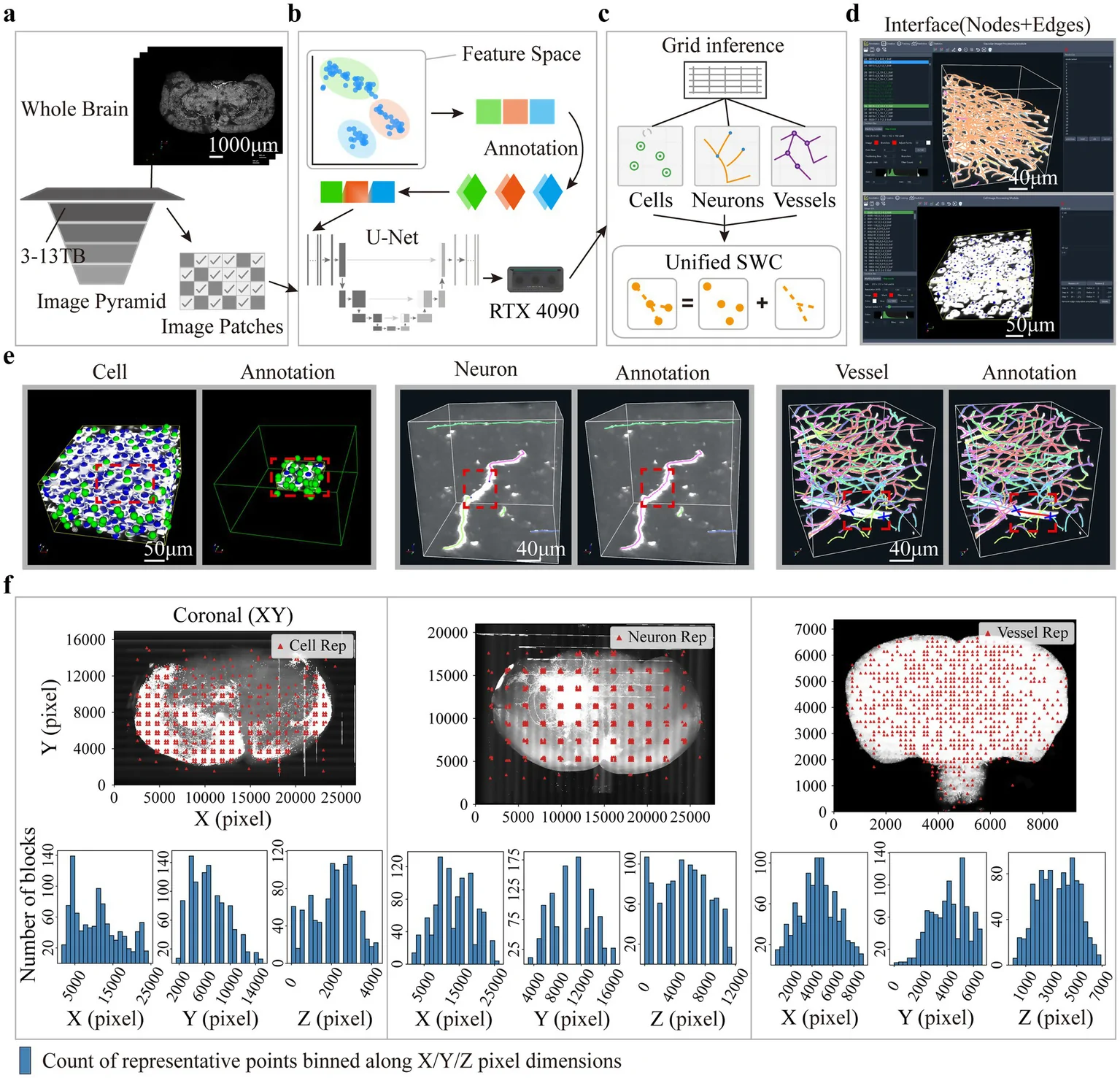
The workflow of BVLab-Analyzer for whole-brain multi-structure segmentation and 3D reconstruction. **(a)** TB-scale raw imaging data (3–13 TB) are processed through image pyramid construction and grid-based block tiling. **(b)** Supervised U-Net training pipeline: K-Means clustering of image features identifies distinct biological signal clusters. From each cluster, 1–2 representative image blocks are selected, manually inspected, and then semi-automatically annotated to generate the training labels. A standard U-Net is trained on an RTX 4090 GPU using these annotations. **(c)** Grid-based parallel inference: the brain volume is partitioned into uniform blocks, and three target structures, somas, neurons, and vasculature, are predicted in parallel, with all results fused into a unified SWC file. **(d)** Software interface: upper panel shows 3D vascular rendering, lower panel shows cell soma reconstruction (node-only). **(e)** Manual annotation examples for three structures: each column (cells, neurons, vasculature) shows the original 3D raw image (left) and the annotated ground truth (right), with red dashed boxes indicating target regions. **(f)** Spatial distribution of the K-Means-based sampling strategy across the whole brain: the spatial position distribution of the three structure types in the coronal plane (upper), and the statistical distribution of sampling in three-dimensional space (lower).

The software supports batch sequential processing of images within a single project, but does not currently provide a command-line interface or cross-project scripting orchestration. The inference pipeline is fixed to the built-in 3D U-Net: its model-loading logic and the associated GPU memory scheduling, 3D block-wise prediction, and post-processing pipeline are all built around this architecture, so other network structures cannot be directly plugged in. Nevertheless, users can replace the model weight files to load weights of a self-trained 3D U-Net with the same architecture, adapting the software to different datasets or tasks.

The data engine directly supports three mainstream multi-dimensional bioimaging formats: (i) standard multi-page TIFF stacks (8-bit/16-bit); (ii) BioimageVision (BV) format (Fast Loading and Transformation of Large-Volume Bioimaging Data Consistently Approaching the I/O Peak Speed) and (iii) the open OME-Zarr format, featuring cloud-optimized multi-resolution image pyramid structures (Moore et al., 2023). For pyramid-supporting formats (BV and OME-Zarr), the software automatically parses downsampling levels, allowing users to switch between resolution levels (0–5) during visualization.

To address the core challenge of terabyte-scale whole-brain volumetric data far exceeding the memory and GPU memory limits of a single machine, the software implements a comprehensive grid-based block-wise computation architecture with an active clustering sampling mechanism to support stable computation on ultra-large volumetric data.

#### 2.1.1 Adaptive 3D block-wise tiling

The software automatically partitions complete whole-brain 3D data (3–13 TB) into tens of thousands of independent sub-volume blocks, with differentiated block sizes for different analysis tasks: cell detection tasks use 272 × 272 × 144 voxel blocks, while neuronal processes reconstruction and vascular network segmentation uniformly use 192 × 192 × 192 voxel blocks, maximizing compatibility with consumer-grade GPUs such as the RTX 4090 (24 GB VRAM, ~14–16 GB usable during training). Block sizes balance two constraints: GPU memory (all configurations stay within 8–14 GB) and receptive field—cell detection requires larger xy-plane coverage for soma morphology (272 × 272 × 144), while neuron/vessel tracing needs isotropic blocks for z-continuity (192 × 192 × 192). These parameters are adjustable via the Advanced Settings panel; proportional rescaling is advised when image resolution differs from this study.

#### 2.1.2 Clustering-based representative block sampling

To address the prohibitive cost of annotating massive image blocks, the software includes a built-in K-Means clustering algorithm that extracts grayscale histograms and maximum intensity projection features from all sub-blocks for feature-space clustering, producing 1,000–2,000 clusters. From each cluster, the block closest to the cluster center is selected as a candidate, yielding a diverse candidate pool. Researchers then manually inspect and select 100–200 representative blocks from this pool for sparse annotation. This workflow substantially reduces redundant annotation while ensuring coverage of heterogeneous signal patterns. The final training block counts are 166 (cell), 86 (neuron), and 120 (vessel) (Table 2). Selected training blocks are randomly split into training and validation sets at an 8:2 ratio. Training and test blocks are drawn from non-overlapping brain regions, ensuring independent evaluation.

**Table 2:** Processing efficiency metrics for three representative whole-brain datasets.

Metric	Cell detection (16-bit)	Neuron tracing (16-bit)	Vascular segmentation (8-bit)
Whole-brain volume	3.6 TB	12.36 TB	0.47 TB
Dimensions (x/y/z)	26,600/16,949/4,400	27,868/21,000/11,611	9,310/7,386/7,332
Training set preparation	3 min	2 min	4 min
Training blocks	166	86	120
Training duration	2.8 h	1.9 h	2.4 h
Whole-brain prediction	41 h	129 h	6.3 h
Stitching duration	1 min (27.4 MB)	17 min (2.89 GB)	36 min (9.22 GB)
GPU memory (training)	8–14 GB	8–14 GB	8–14 GB

#### 2.1.3 Sliding window inference and seamless stitching

Whole-brain inference employs an overlapping sliding window strategy. After inference, stitching positions are determined based on the overlap region size and the spatial location of each block, and the topological structures of all blocks are merged to reconstruct the topology in the whole-brain coordinate space. For cell detection, the overlap region is set to 32, 32, and 20 voxels in x, y, and z, respectively; for neuron and vascular reconstruction, the overlap region is set to 4, 4, and 2 voxels in x, y, and z, respectively. Overlap sizes follow biological scale: cell (32 × 32 × 20) ≈ 1.5–2 × soma diameter, preventing boundary truncation; neuron/vessel (4 × 4 × 2) covers several fiber diameters, ensuring continuity with minimal redundancy. These parameters are also adjustable via Advanced Settings, with proportional rescaling for different pixel resolutions.

#### 2.1.4 Long-duration computation interrupt and resume mechanism

To handle inference durations of tens of hours for terabyte-scale data, the software records completed sub-volume computation logs in real time. In the event of unexpected interruptions such as power outages, system restarts, or temporary workstation occupation, the software can automatically skip already-processed blocks and resume remaining tasks without recomputation from scratch, perfectly adapting to shared workstation usage scenarios in laboratories.

#### 2.1.5 Storage, I/O, and interactive visualization

For large volumetric datasets (the largest in this work is approximately 12.36 TB), we recommend storing the dataset being processed on a local SSD for better I/O performance. NAS-mounted directories can also be used as input paths, but inference throughput decreases as network bandwidth decreases, depending on network bandwidth, block size, and read pattern.

Users can specify a level parameter to run inference at a chosen resolution level, balancing accuracy and throughput. Prediction results are precomputed and stored: after whole-brain inference, segmentation masks and skeletons are saved as .tif and .swc files, respectively. Visualization is implemented in VTK, allowing users to browse raw images and segmentation results in the viewer, with real-time interactive rotation, zooming, and panning.

### 2.2 Smart annotation and training

To minimize manual labor costs, BVLab-Analyzer provides customized interactive Smart Annotation strategies for different morphological types.

#### 2.2.1 Sub-volume isolation

For processing dense, overlapping signal clusters in thick tissue, users can define compact 3D bounding boxes. The software dynamically crops voxels outside the box to eliminate visual background interference, without performing destructive data cropping on disk (Zhou et al., 2021).

#### 2.2.2 Cell annotation

For point-like structures, a single click at the soma center registers the 3D coordinate (< 1 s), without requiring contour or bounding box segmentation. Point annotations are directly saved to the SWC topology matrix as independent root nodes (structure type 1, parent node −1) (Cannon et al., 1998).

#### 2.2.3 Neuron and vascular skeleton annotation

For linear structures, users place sparse anchor points along the fiber path. The software automatically generates intermediate path coordinates along 3D line segments and establishes parent–child connections compliant with the SWC specification. Neuron and vascular skeletons are represented as complete skeleton trees (type = 2), with node radii computed by Euclidean distance transform and disconnected components identified as independent root nodes. Users can directly fine-tune nodes or perform branch operations (merge, disconnect, or batch delete fragmented segments) within the browsing interface.

#### 2.2.4 Coarse vessel ray-casting annotation

Large-diameter vessels often exhibit weak signals or cavities due to clearing or staining characteristics. In this mode, the system projects orthogonal rays outward from user-interactively placed reference points. Ray endpoints detect local intensity drops at the vessel wall boundary, recording the outer wall coordinates and then automatically computing the true cross-sectional midpoint and local radius (Du et al., 2026). After annotation, the software automatically converts sparse annotations into voxel masks for neural network training: cell points are expanded into local Gaussian blobs, and skeleton trajectories are convolved with Gaussian kernels to form continuous tubular volumes. This training strategy bypasses the need for labor-intensive dense 3D annotation, focusing instead on cell locations and skeletal trajectories. Importantly, once the cell bodies and skeletons are obtained, morphological features can be automatically computed (Li et al., 2020), further enriching the analytical output. The effectiveness of this sparse-to-dense strategy has been validated in previous studies (Du et al., 2026).

### 2.3 Block-wise inference and post-processing of segmentation results

All three segmentation and reconstruction tasks share a common 3D U-Net backbone architecture (Ronneberger et al., 2015; Çiçek et al., 2016), with independent weights trained separately for cells, neurons, and vasculature, using unified training hyperparameters: Adam optimizer, initial learning rate of 1 × 10–4, batch size of 4–8, Soft Dice loss function, combined with a 100-epoch patience early stopping strategy and a maximum of 500 training epochs to prevent overfitting. The training interface displays loss curves and validation set prediction previews in real time, supporting pause and checkpoint resumption at any time. On RTX 4090 hardware, single-task complete training duration is controlled within 1.9–2.8 h, with GPU memory usage stably maintained at 8–14 GB, fully compatible with consumer-grade GPU compute capacity.

After model training, the software executes whole-brain block-wise parallel inference using the grid-based block engine, with differentiated post-processing algorithms designed for three target structure types. All output results are uniformly adapted to the neuroscience community-standard SWC topology format, completely eliminating data format barriers caused by multi-tool workflows.

*Soma detection*: threshold segmentation and connected component labeling are applied to the network output probability map, automatically extracting all soma centroid coordinates and directly outputting independent SWC node files, completing whole-brain quantitative cell counting.

*Neuron reconstruction*: after binarizing the probability map, the PointTree algorithm is applied for continuous tracing axons and dendrites (Cai et al., 2025), maximizing neural fiber tracing accuracy and reducing the manual revision workload for loop reconstruction.

*Vascular network extraction*: the Lee 3D thinning algorithm is used to extract vascular centerline skeletons (Lee et al., 1994), combined with Euclidean distance transform to compute real-time vessel diameters for each segment, reconstructing the complete reticular microvascular topology.

Additionally, the software includes a built-in one-stop morphological statistics module that can automatically batch-compute key quantitative indicators such as cell spatial density, total neural fiber length, branch point count, fiber bifurcation angle, and vessel diameter distribution. All statistical results can be directly exported as standard CSV tables for immediate use in downstream scientific chart plotting and data analysis.

### 2.4 End-to-end complete workflow

Leveraging the full suite of software modules described above, BVLab-Analyzer forms a closed-loop, zero-code, fully automated TB-scale whole-brain image analysis pipeline, completed entirely within a single software package (as shown in Figures 1a–c): Step 1:import multi-format ultra-large whole-brain data, with automatic image pyramid construction and grid-based block partitioning; Step 2:select representative image blocks via K-Means clustering, combined with Smart Annotation tools for rapid sparse annotation and model training; Step 3:load trained weights to execute whole-brain block-wise inference, automatically completing result stitching and topology repair; Step 4:perform structure-specific post-processing, uniformly outputting SWC topology files and quantitative statistical tables, simultaneously achieving cell detection, neuron reconstruction, and vascular segmentation across all three analysis tasks.

### 2.5 Skeleton-based unified evaluation framework

To evaluate the segmentation performance across all three structural types: cells, neurons, and vessels, we adopt a unified skeleton-based metric framework. For each structure, the network prediction generates a set of skeleton points P (or cell centers for cell detection), while the ground-truth annotation provides the corresponding reference set G. The evaluation is based on point-wise matching within a predefined Euclidean distance threshold τ = 3 voxels. Specifically, a predicted point p ∈ P is considered a true positive if the distance to its nearest point in G is less than τ; otherwise, it is classified as a false positive. Conversely, a ground-truth point g ∈ G with no predicted point within τ is counted as a false negative. Based on these definitions, we compute recall = TP/(TP + FN), precision = TP/(TP + FP), and the F1-score = 2 × (precision × recall)/(precision + recall) for each structural category. This evaluation paradigm is directly applicable to all three tasks: for cell detection, the “skeleton points” correspond to annotated cell centers; for neuron tracing and vascular skeleton reconstruction, they correspond to the centerline points of axonal/dendritic processes and vascular trees, respectively. The unified metric framework ensures consistent and quantitative cross-structural comparison of algorithmic performance (Li et al., 2025).

To prevent spatial leakage, the dataset partitioning follows a region-isolated, within-brain independent split strategy: training and test blocks are drawn from spatially non-overlapping regions of the same brain, eliminating spatial dependence at the architectural level rather than relying solely on per-block distance thresholds. Test blocks were extracted from a large independent data block (~10 GB) spatially separated from training regions. A uniform spatial grid initialized candidate points, and image features (mean, variance, gradient entropy) were clustered via K-Means to balance selection across heterogeneous signal patterns, with the sampling distribution illustrated in Figure 1f. A hard spatial constraint of ≥ 64 voxels between any test and training block center eliminated spatial autocorrelation. Approximately 5–15% of blocks are empty/background to reflect the true whole-brain distribution; primary metrics are computed only on signal-containing blocks (~1,000 per task) to avoid 0/0 ambiguity. The protocol is summarized in Table 3.

**Table 3:** Test set construction summary.

Dimension	Cell detection	Neuron tracing	Vascular segmentation
Data source	Independent ~10 GB block, anatomically separate from training	Same	Same
Sampling strategy	Uniform grid + K-Means feature-balanced selection	Same	Same
Min. training–test distance	≥ 64 voxels	≥ 64 voxels	≥ 64 voxels
Signal blocks	~1,000	~1,000	~1,000
Background blocks	~5–15%	~5–15%	~5–15%
Cross-specimen	No (see discussion)	No	No

### 2.6 Algorithmic comparison with existing methods

To clarify the relationship between BVLab-Analyzer and existing tools at the algorithmic level, we decompose the processing pipeline into individual components and compare each with established methods (Table 4).

**Table 4:** Algorithmic component comparison.

Component	Relationship	Description
3D U-Net backbone	Reused	Classic architecture (Ronneberger et al., 2015; Çiçek et al., 2016), same foundation as nnU-Net, Cellfinder, and TrailMap; trained with independent weights per structure type
K-Means clustering-based block selection	New	First use of unsupervised clustering for TB-scale training set selection; extracts histogram + MIP features, clusters into 1,000–2,000 groups, selects one representative block per cluster. Replaces experience-based random selection in existing tools, reducing annotation from hundreds of blocks to 86–166
Sparse-to-dense annotation conversion	New	Cell points → Gaussian blobs; skeleton lines → tubular Gaussian kernels. Distinct from contour-based (Cellpose/StarDist), pixel-level (ilastik), and per-neuron tracing (Vaa3D/BigNeuron) paradigms; substantially reduces annotation labor
Block-wise inference with sliding window	Improved	Similar to nnU-Net patch-based inference, but structure-specific: cell uses anisotropic 272 × 272 × 144 (matching soma xy-plane receptive field), neuron/vessel use isotropic 192 × 192 × 192 (ensuring z-direction continuity). Existing tools use uniform patch sizes
Overlap stitching	Improved	Structure-differentiated: cell 32 × 32 × 20 (~1.5–2 × soma diameter), neuron/vessel 4 × 4 × 2 (sufficient for linear structures). Minimizes redundant computation vs. fixed-overlap approaches (nnU-Net)
PointTree neuron tracing	Reused	Cai et al. (2025); extracts continuous axonal/dendritic skeletons from probability maps, unlike TrailMap which outputs only probability maps
Lee 3D thinning for vascular skeletonization	Reused	Lee et al. (1994); centerline extraction + Euclidean distance transform for diameter, consistent with HP-VSP
Connected component analysis for soma extraction	Standard	Thresholding + labeling + centroid calculation, consistent with ClearMap and Cellfinder
Unified SWC topology encoding	New	Unifies three structural topologies into standard SWC (cell → root nodes, neuron → skeleton tree, vessel → skeleton tree + radius). Existing tools output heterogeneous formats (CSV, probability maps, instance masks, segmentation masks), creating inter-tool format barriers

Three algorithmic contributions emerge: (1) clustering-driven training set construction, replacing manual selection with data-driven sampling, reducing annotation volume by ~10×; (2) automated sparse-to-dense annotation conversion, requiring only key topological points/lines rather than pixel-level contours; (3) structure-adaptive block-wise inference, with differentiated parameters for distinct morphologies under shared GPU constraints.

## 3 Results

### 3.1 Whole-brain data description and processing efficiency

Three representative whole-brain datasets covering distinct biological structures and imaging modalities were used: a 3.6 TB, 16-bit c-Fos immunofluorescence-labeled cell detection volume (26,600 × 16,949 × 4,400 voxels); a 12.36 TB, 16-bit GFP-labeled neuron tracing dataset (27,868 × 21,000 × 11,611 voxels); and a 0.47 TB, 8-bit DyLight 594-labeled vascular segmentation volume (9,310 × 7,386 × 7,332 voxels). These datasets spanned three orders of magnitude in data volume (0.47–12.36 TB) and encompassed both 8-bit and 16-bit imaging depths, collectively covering cell detection, neuron tracing, and vascular segmentation tasks (Table 2). All processing was performed on a single workstation equipped with one RTX 4090 GPU (24 GB VRAM), a 32-core CPU, 64 GB RAM, and Windows 11.

Training set preparation via K-Means clustering completed within 2–4 min across all datasets, yielding 86–166 training blocks. Single-GPU training required 1.9–2.8 h with GPU memory consistently maintained at 8–14 GB. Whole-brain prediction time scaled with data volume from 6.3 h (0.47 TB) to 129 h (12.36 TB), with stitching overhead remaining negligible (1–36 min, Table 2). The interrupt-and-resume mechanism was validated on the neuron dataset: prediction was paused after 48 h of runtime, the workstation was used for daily office work during one business day, and prediction was subsequently resumed with completed sub-volumes unaffected. This confirms that the interrupt-and-resume mechanism effectively allows long-running inference tasks to be paused and resumed without loss of progress, enabling the same workstation to serve both computation-intensive analysis and routine daily use.

### 3.2 Whole-brain cell detection performance evaluation

To assess the automated quantification performance of the proposed algorithm in high-throughput, large-volume imaging contexts, we used a representative cell detection dataset featuring marked regional heterogeneity in cell distribution and substantial signal-to-background intensity fluctuations.

As shown in Figure 2a, the proposed algorithm demonstrated robust cross-scale three-dimensional spatial localization capability. From the macroscopic whole-brain view (Figure 2a, left) progressively zooming in to microscopic sub-volumes where individual cells are clearly visible (Figure 2a, right), the algorithm’s automatically detected and labeled cell centers (orange pseudo-colored dots) showed high consistency with the spatial distribution of cells in the original grayscale images. The whole-brain prediction model was derived from our constructed training set, which contained data blocks with large cell density differences. In these data blocks, we first annotated the spatial positions of cells, then derived a Gaussian kernel centered at each position as its mask for network training (Figure 2b).

**Figure 2:**
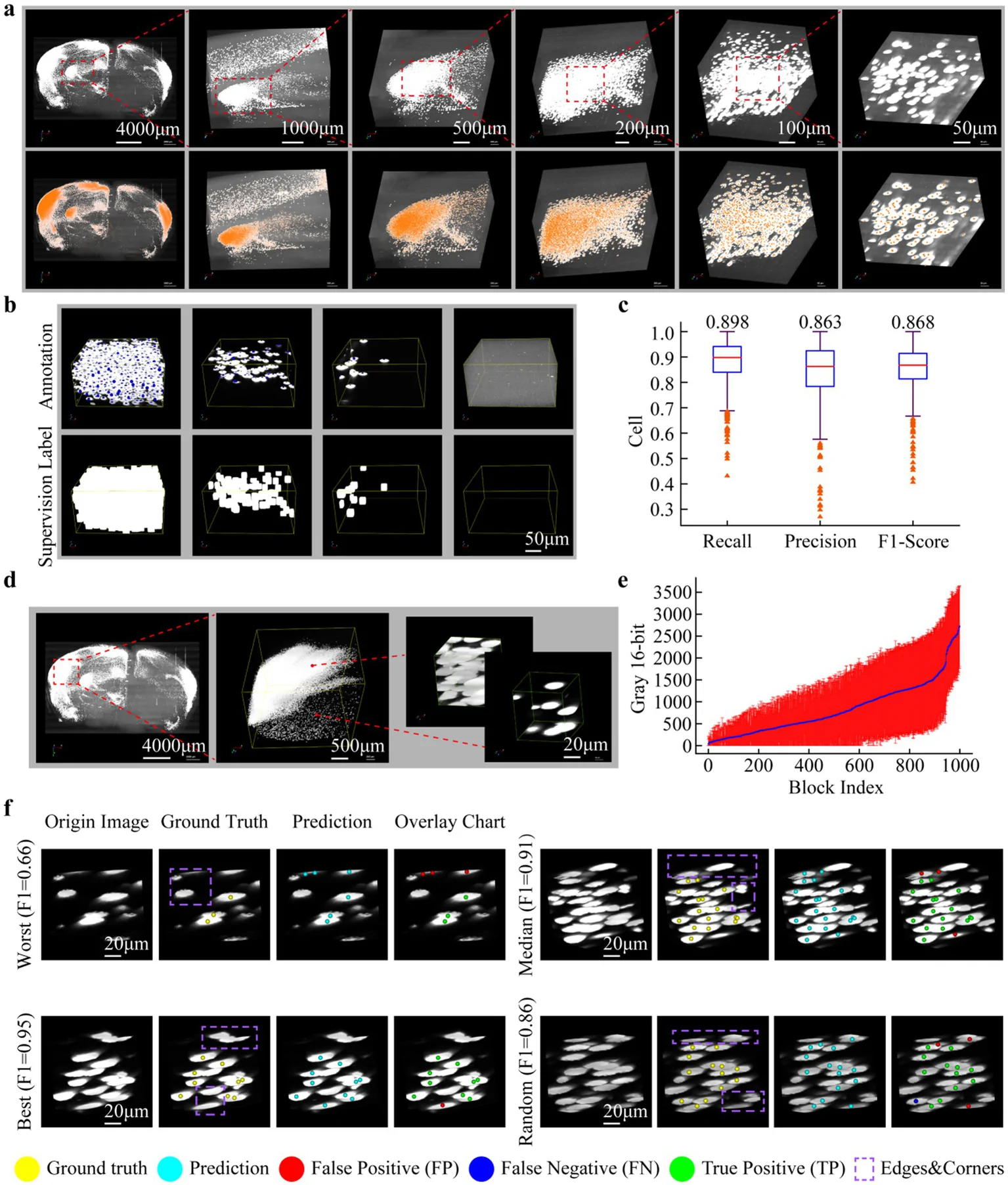
Soma detection performance. **(a)** Multi-scale visualization of somas detection: upper row, original 3D brain volume with red dashed boxes indicating progressively zoomed-in regions; lower row, predicted cell centers (orange) overlaid on original images. **(b)** Training data: manually annotated cell positions (upper) and corresponding Gaussian-blob supervision labels (lower). **(c)** Box plots of Recall, Precision, and F1-score across 1,000 test sub-blocks (64 × 64 × 64 voxels), with median values above each plot and orange triangles representing individual sub-block scores. **(d)** Test sub-block extraction: a ~ 10 GB raw data block is cropped from the whole brain, from which 1,000 independent sub-blocks are extracted. **(e)** Grayscale statistics of all test sub-blocks: x-axis, block index; y-axis, 16-bit grayscale; red shading, voxel range per block; blue curve, mean grayscale trend. **(f)** Fine-grained comparison of cell detection: four sample groups (best, median, worst and random) respectively display the raw image, ground truth, prediction result, and overlay comparison.

To provide quantifiable support for the algorithm’s reliability, we randomly selected nearly one thousand independent 3D image blocks from a large data block within this brain and performed statistical analysis of the detection results (Figure 2c). Quantitative evaluation indicated that the missing detection rate was suppressed to a very low level; meanwhile, the median precision reached 0.863, effectively controlling false positive detections; ultimately, the comprehensive evaluation metric F1-score reached 0.868. Although the presence of a few challenging image blocks resulted in some low-score outliers in the statistical distribution (orange triangles in Figure 2c), the overall compactness of the distribution confirmed the high statistical stability and accuracy of this automated quantification pipeline.

Figures 2d,e demonstrate the model’s performance in the face of severe signal non-uniformity. In the data used to evaluate our model (Figure 2d), among 1,000 randomly sampled test sub-volumes (Figure 2e), the 16-bit grayscale mean (blue curve) of each image block exhibited a large-span linear stepwise distribution from several hundred to nearly 3,000, and the intra-block voxel value range (red shading) fluctuated substantially. However, the high-precision performance in Figure 2c confirms that even under substantial grayscale dynamic range fluctuations and strong background heterogeneity interference, the algorithm can adaptively maintain high-level detection output without complex preprocessing. This result supports the application value of the algorithm in processing real-world large-scale, imperfect microscopic imaging datasets.

Figure 2f further presents a fine-grained comparison of cell detection, showing the raw image, ground truth, prediction result, and overlay comparison for each group. The Best case (F1 = 0.95) shows well-separated cells with exact predictions; the Median case (F1 = 0.91) exhibits typical performance with occasional boundary blur; the Worst case (F1 = 0.66) presents sparse cell clusters with boundary errors; and the Random case (F1 = 0.86) reflects the general performance of a random sample.

### 3.3 Whole-brain neuron morphological tracing performance evaluation

To further validate the software’s performance on complex, low-SNR, and strongly anisotropic neuronal fiber networks, we evaluated it on a whole-brain-scale neuron dataset. As shown in Figure 3a, the algorithm demonstrated robust cross-scale neuronal morphological tracing and reconstruction capability. From the macroscopic whole-brain view of complex neural fiber projections (Figure 3a, left), progressively focusing and zooming in to microscopic sub-volumes containing individual neuronal somata and their densely interwoven dendritic and axonal branches (Figure 3a, right), the algorithm’s automatically traced and extracted neural topology (orange pseudo-colored regions) showed high spatial correspondence with the fiber trajectories in the original images. Due to the strong directional anisotropy of neural fibers in space, we deliberately selected four representative 3D sub-volumes with different fiber intersection densities for detailed validation in the training set (Figure 3b). These data types include: regions with densely fiber entanglement and severe crossing overlap, and background noise regions containing only discrete weak signals or non-target halos. The appropriate training set construction provided high accuracy for whole-brain-scale reconstruction.

**Figure 3:**
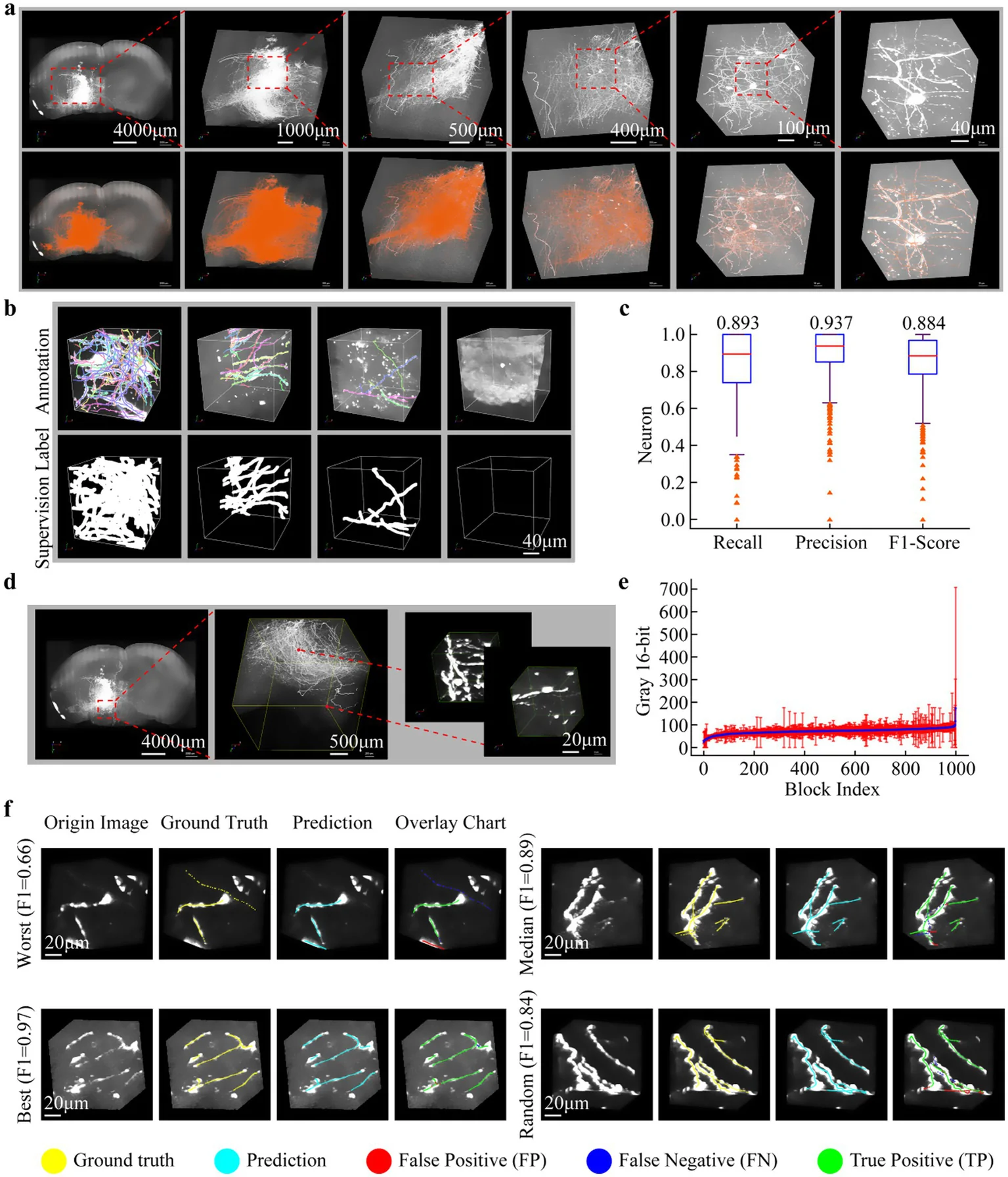
Neuron reconstruction performance. **(a)** Multi-scale visualization: whole-brain GFP-labeled neuronal fibers (upper, original) with predicted skeletons (lower, orange) overlaid, red boxes mark zoom-in regions. **(b)** Training data: manually traced 3D neuronal skeletons (upper, multi-color) and corresponding supervision labels (lower). **(c)** Box plots of Recall, Precision, F1-score across 1,000 test sub-blocks (64^3^ voxels), median values 0.893, 0.937, 0.884; orange triangles are individual scores. **(d)** Test sub-block extraction: ~10 GB data block cropped from whole brain, yielding 1,000 independent sub-blocks. **(e)** 16-bit grayscale statistics: x-axis block index; y-axis intensity; red shading voxel range; blue curve mean trend. **(f)** Fine-grained comparison of neuron tracing: four sample groups (best, median, worst and random) respectively display the raw image, ground truth, prediction result, and overlay comparison.

Quantitative tracing metrics and statistical robustness. Quantitative statistical analysis of approximately 1,000 independent test 3D sub-volumes (Figure 3c) further quantified the consistent performance of this automated pipeline. Experimental results showed that the algorithm achieved a median recall of 0.893 on the test set, effectively curbing breakage and missed tracing of slender neuronal branches in low signal-to-noise ratio environments; meanwhile, the median precision reached a high level of 0.937, indicating effective discrimination against background impurities and non-specific fluorescent artifacts, reducing false positive tracing; the final comprehensive evaluation metric F1-score was 0.884. Although complex local neural structures and dense intertwining caused some particularly challenging image blocks to appear as low-score outliers in the statistical distribution (orange triangles in Figure 3c), the metrics for the vast majority of image blocks remained closely concentrated toward the median, demonstrating statistical robustness in whole-brain-scale data processing.

Performance against weak fluorescence and drastic intensity heterogeneity. In whole-brain light-sheet microscopy imaging, neural fibers often exhibit very low image contrast accompanied by drastic local intensity fluctuations as an underlying physical challenge. Figures 3d,e demonstrate the algorithm’s robust performance in addressing such signal non-uniformity challenges. Among the 1,000 tested sub-volumes (Figure 3e), the 16-bit grayscale mean (blue curve) was generally suppressed to a low brightness range below 100, and the intra-block voxel value range (red shading) exhibited drastic fluctuations in edge regions. However, combined with the high-precision segmentation metrics in Figure 3c, it can be confirmed that even under stringent ultra-low contrast backgrounds, weak and intermittent fiber signals, and localized sudden strong noise interference, we can still adaptively capture the continuous deformation and topological orientation of neurons without complex preprocessing, providing technically valuable support for whole-brain-scale single-neuron complete morphological reconstruction and neural circuit quantitative analysis.

Figure 3f further presents a fine-grained comparison of neuron tracing, showing the raw image, ground truth, prediction result, and overlay comparison for each group. The Best case (F1 = 0.97) shows complete tracing without breaks in a sparse fiber scenario; the Median case (F1 = 0.89) represents typical medium-density fiber tracing with good performance; the Worst case (F1 = 0.66) exhibits fiber breaks in an extremely low-SNR region, reflecting the algorithm’s performance boundary under extreme conditions; and the Random case (F1 = 0.84) provides an unbiased visual reference for the overall performance.

### 3.4 Whole-brain vascular reconstruction performance evaluation

Here, we comprehensively evaluated our software using the whole-brain-scale vascular dataset as a benchmark. As shown in Figure 4a, the algorithm demonstrated cross-scale vascular spatial tracing capability. From the macroscopic whole-brain vascular network view (Figure 4a, left) progressively focusing and zooming in to densely interconnected microscopic microvascular sub-volumes (Figure 4a, right), the algorithm’s automatically segmented and extracted skeletons and boundaries (orange pseudo-colored regions) showed high consistency with the complex vascular trees in the original images. We present typical data from the training set. These data include regions with densely intertwined and multi-branch crossing vascular networks (Figure 4b, leftmost column), and regions with very few fine vessels and vessel-free background noise (Figure 4b, rightmost column). The training masks follow the vessel centerline, with higher grayscale values at the center and lower values away from the center.

**Figure 4:**
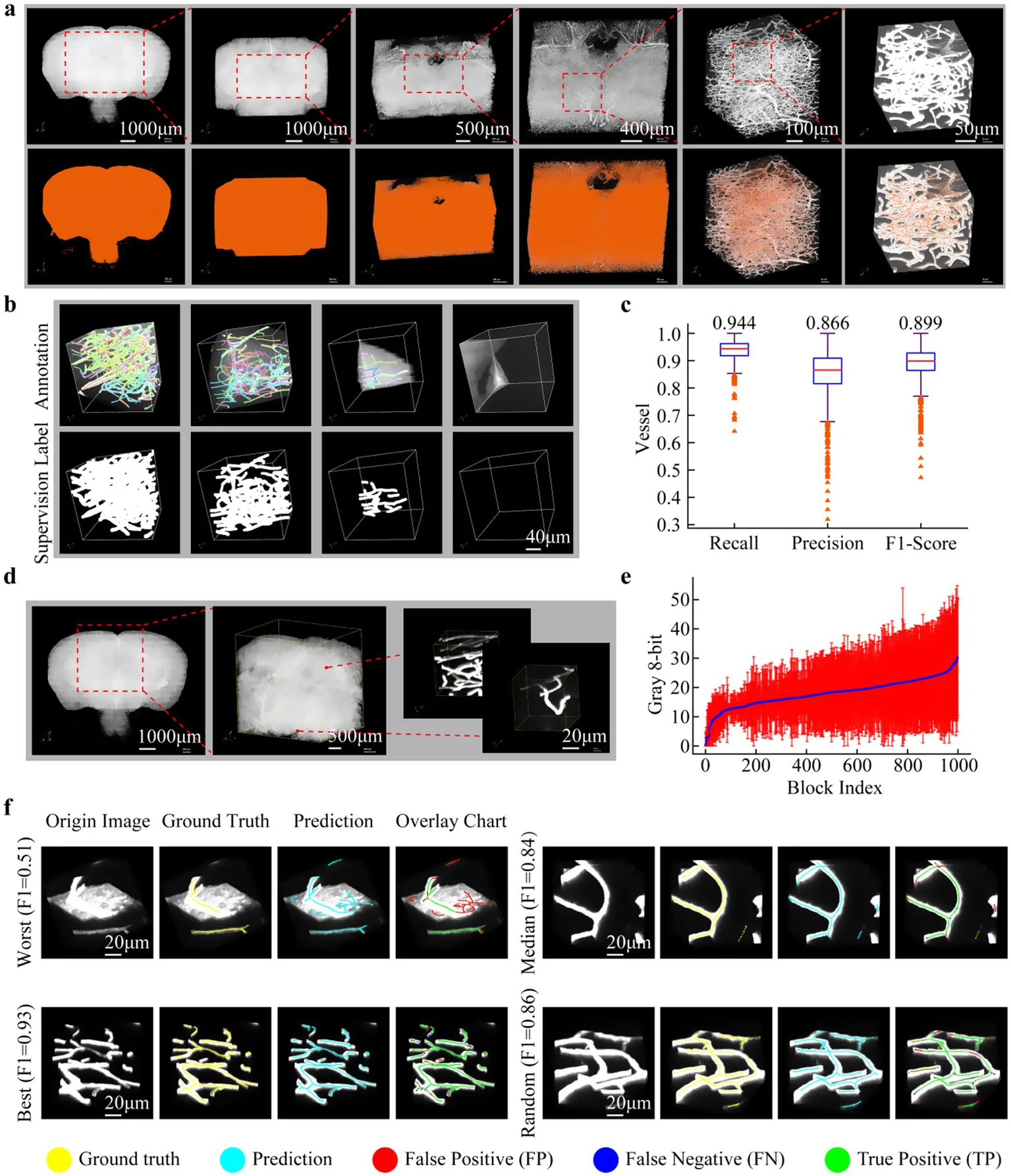
Vascular reconstruction performance. **(a)** Multi-scale visualization: whole-brain DyLight 594-labeled vasculature (upper, original) with predicted vascular skeletons (lower, orange) overlaid, red boxes mark zoom-in regions. **(b)** Training data: manually annotated 3D vascular skeletons (upper, multi-color) and corresponding supervision labels (lower). **(c)** Box plots of Recall, Precision, F1-score across 1,000 test sub-blocks (64^3^ voxels), median values 0.944, 0.866, 0.899; orange triangles are individual scores. **(d)** Test sub-block extraction: ~10 GB data block cropped from whole brain, yielding 1,000 independent sub-blocks. **(e)** 8-bit grayscale statistics: x- axis block index; y-axis intensity; red shading voxel range; blue curve mean trend. **(f)** Fine-grained comparison of vessel reconstruction: four sample groups (best, median, worst and random) respectively display the raw image, ground truth, prediction result, and overlay comparison.

Quantitative statistical evaluation (Figure 4c) further confirmed the reliability and high precision of this automated quantification pipeline. In testing nearly one thousand independent 3D image blocks from one data block, the model achieved a median recall of 0.944, indicating effective control of breakage and missed detection in continuous fine vessels; the median precision was 0.866, effectively suppressing false noise and background artifact misclassifications; the final comprehensive evaluation metric F1-score reached 0.899. Although a few particularly challenging low signal-to-noise ratio edge blocks resulted in some low-score outliers (orange triangles in Figure 4c), the overall metric distribution was closely concentrated toward the median, demonstrating strong statistical stability.

Performance against ultra-low contrast and strongly non-uniform backgrounds. Unlike cell images, large-volume whole-brain vascular imaging (especially 8-bit depth data) is often accompanied by low image contrast and strong background fluctuation challenges. Figures 4d,e demonstrate the model’s robust performance against such underlying physical noise. Among the 1,000 sampled test sub-volumes (Figure 4e), the 8-bit grayscale mean (blue curve) of image blocks was generally in the low range of 10 to 30, and the intra-block voxel value range (red shading) appeared pronounced relative to the signal itself. However, combined with the strong segmentation results in Figure 4c, it can be concluded that even under such stringent ultra-low contrast backgrounds, complex topological intertwining, and strong noise interference, the algorithm still exhibits reliable feature extraction capability, maintaining high-quality continuous vascular skeletons without complex preprocessing. This is valuable for whole-brain-scale microvascular connectivity and morphological quantitative analysis.

Figure 4f further presents a fine-grained comparison of vessel reconstruction, with each group displaying the raw image, ground truth, prediction result, and overlay comparison. The Best case (F1 = 0.93) corresponds to a clear vascular network with complete reconstruction of both main trunks and branches; the Median case (F1 = 0.84) represents typical performance with good overall structural preservation; the Worst case (F1 = 0.51) involves a large-vessel region where internal false positives appear due to the cavity effect, reflecting the algorithm’s limitations in luminal structures; and the Random case (F1 = 0.86) reflects the general reconstruction performance of a randomly selected sample.

## 4 Discussion

BVLab-Analyzer addresses key practical barriers in TB-scale whole-brain volumetric analysis with four core strengths. First, it enables stable full-process analysis of up to 12 TB whole-brain data using only a single consumer-grade GPU. Powered by adaptive volumetric tiling and optimized GPU memory scheduling, the software eliminates the reliance on expensive multi-GPU clusters required by most existing neuroimaging tools, making large-volume brain quantification accessible for ordinary laboratories and public imaging facilities with limited computational resources.

Second, this toolkit integrates cell detection, neuron reconstruction, and vascular skeletonization into one unified GUI pipeline. To our knowledge, no existing software supports concurrent analysis of all three core neurovascular component. The unified workflow avoids format incompatibility and spatial registration errors caused by switching multiple separate tools, supporting direct correlation analysis of neuronal morphology and vascular topology via standardized SWC outputs.

Third, built-in annotation tools and optional clustering-based sampling reduce manual workload. The Smart Annotation module cuts manual labeling time by 50–70%. The embedded K-Means clustering module selects representative image blocks, reducing required training annotation volume by nearly one order of magnitude and avoiding redundant labeling on visually similar background regions.

Fourth, the integrated interrupt-and-resume mechanism solves a critical pain point for long-duration computation. The longest whole-brain inference lasts 129 h, and unexpected interruptions are common on shared workstations. This module records block-wise computation logs and resumes tasks accurately without repeated calculation, improving workstation utilization in daily laboratory scenarios.

We chose a standalone desktop application rather than a Fiji/napari plugin (Schindelin et al., 2012; Sofroniew et al., 2025) or a web/server solution for three reasons. (1) Physical constraints of TB-scale data: uploading a 12 TB dataset to a server requires ≈33 h at a 100 MB/s bandwidth, and gigabit NAS read access becomes a severe I/O bottleneck; a local application reading directly from a PCIe 4.0 NVMe SSD (≈7 GB/s) is the only practical option. (2) Zero-code usability: a standalone executable bypasses Python environment configuration and CUDA version compatibility issues, a substantially lower barrier than platforms requiring host installation and plugin configuration. (3) End-to-end optimization: TB-scale inference demands fine-grained GPU memory scheduling, block-wise strategies, and cross-module interrupt-and-resume, all of which rely on unified project-file management and internal state coordination that generic plugin frameworks cannot provide. The standalone design remains compatible with the broader ecosystem—exported SWC files can be opened directly in Vaa3D (Peng et al., 2010) and neuTube (Feng et al., 2015), CSV statistics can be used in any analysis environment, and a Python API is planned for a future release.

From the algorithmic perspective, three contributions distinguish BVLab-Analyzer: clustering-driven training set construction (~10 × annotation reduction), automated sparse-to-dense annotation conversion, and structure-adaptive block-wise inference. In the broader tool ecosystem, BVLab-Analyzer occupies a complementary niche—general-purpose tools (nnU-Net, Cellpose) deliver high single-task accuracy but require user expertise for pipeline assembly, while specialized tools (ClearMap, Cellfinder, TrailMap) target single structures without unified multi-type analysis. BVLab-Analyzer bridges these gaps by integrating three pipelines within a single zero-code GUI, with unified SWC output enabling direct cross-structure correlation—particularly suited for neurovascular coupling studies.

The unified framework provides practical advantages beyond single-task accuracy: shared spatial coordinates eliminate cross-tool registration errors, and unified SWC output with consistent parameters removes format conversion barriers common in multi-tool workflows. A current limitation is that each channel (cell, neuron, vessel) is processed independently by a dedicated model—the software cannot separate mixed signals within a single channel, a constraint shared by existing tools and a direction for future work.

To contextualize these results, we reference representative whole-brain analyses from independent groups. For cell detection: ACE F1 = 0.75 ± 0.08 (Attarpour et al., 2025), DELiVR F1 = 0.79 (Kaltenecker et al., 2024), SMARTTR F1 = 0.85 (Jin et al., 2025). For neuron tracing: TrailMap recall = 0.752 (Friedmann et al., 2020), D-LMBmap median Dice = 0.90–0.95 (Li et al., 2023), pixel embedding F1 = 0.855 (Fu et al., 2025). For vascular segmentation: VesSAP F1 = 0.84 ± 0.05 (Todorov et al., 2020), VpbSD Dice = 0.852 (Lin et al., 2024), DeepVess Dice = 0.82 (Haft-Javaherian et al., 2019). Our results (cell F1 = 0.868, neuron F1 = 0.884, vessel F1 = 0.899) are at comparable levels. Different biological applications demand different precision: connectome reconstruction requires near-perfect tracing beyond any automated method, while population-level analyses (regional cell counts, vessel density) tolerate localized errors. BVLab-Analyzer is positioned as a high-quality initial segmentation that accelerates manual revision; the built-in interactive proofreading tools (probability map overlay, confidence-ranked review, SWC node editing) are designed for this workflow.

Meanwhile, this software still has limitations. It currently only supports static ex vivo rodent whole-brain data and is incompatible with dynamic *in vivo* imaging and primate large brain datasets. The current implementation is also limited to Windows platforms, which may restrict adoption in laboratories using Linux-based computing clusters. In future updates, we will optimize pipeline compatibility, add batch processing functions to expand applicable scenarios, and explore cross-platform support.

For the low-scoring outlier regions shown in the box plots (e.g., dense cell clusters, extremely low-SNR fibers, and large-vessel luminal cavities), the current version does not yet provide a mechanism to automatically detect or improve segmentation quality; these regions rely on manual review through the built-in interactive proofreading tools (probability-map overlay and SWC node editing). We plan to introduce uncertainty estimation and adaptive post-processing in future releases to target these difficult regions.

Because the software processes the whole-brain 3D volume end-to-end through fixed-size grid-based block inference (with redundant boundaries to avoid stitching artifacts) and does not rely on anatomical region partitioning or brain atlas annotation, cross-regional and cross-specimen generalization cannot be directly concluded from the current workflow; these require partition-holdout evaluation on atlas-annotated independent datasets.

In conclusion, BVLab-Analyzer provides a lightweight, stable, and all-in-one solution for routine TB-scale whole-brain analysis. It lowers hardware barriers, unifies fragmented analytical workflows, and reduces manual annotation costs, catering to the practical demands of most neuroscience laboratories. This open-access GUI tool complements existing neuroimaging pipelines and facilitates convenient, reproducible whole-brain morphological research.

## Data Availability

The datasets presented in this study can be found in online repositories. The names of the repository/repositories and accession number(s) can be found in the article/supplementary material.
